# Dual-color-emitting green fluorescent protein from the sea cactus *Cavernularia obesa* and its use as a pH indicator for fluorescence microscopy

**DOI:** 10.1002/bio.2497

**Published:** 2013-03-06

**Authors:** Katsunori Ogoh, Takashi Kinebuchi, Mariko Murai, Takeo Takahashi, Yoshihiro Ohmiya, Hirobumi Suzuki

**Affiliations:** a Corporate Research and Development Center, Olympus CorporationKuboyama 2-3, Hachioji, Tokyo, 192-8512, Japan; b Biomedical Research Institute, National Institute of Advanced Industrial Science and TechnologyHigashi 1-1-1, Tsukuba, Ibaraki, 305-8561, Japan

**Keywords:** sea cactus, GFP, dual-color emission, pH indicator, fluorescence microscopy

## Abstract

We isolated and characterized a green fluorescent protein (GFP) from the sea cactus *Cavernularia obesa*. This GFP exists as a dimer and has absorption maxima at 388 and 498 nm. Excitation at 388 nm leads to blue fluorescence (456 nm maximum) at pH 5 and below, and green fluorescence (507 nm maximum) at pH 7 and above, and the GFP is remarkably stable at pH 4. Excitation at 498 nm leads to green fluorescence (507 nm maximum) from pH 5 to pH 9. We introduced five amino acid substitutions so that this GFP formed monomers rather than dimers and then used this monomeric form to visualize intracellular pH change during the phagocytosis of living cells by use of fluorescence microscopy. The intracellular pH change is visualized by use of a simple long-pass emission filter with single-wavelength excitation, which is technically easier to use than dual-emission fluorescent proteins that require dual-wavelength excitation. Copyright © 2013 John Wiley & Sons, Ltd.

## Introduction

Many species of marine organisms are bioluminescent 1, and the molecular basis of bioluminescence has been studied extensively in numerous species of the phylum Cnidaria. Bioluminescence of these species is due to chemical reaction of the substrate coelenterazine (a luciferin) with a luciferase or photoprotein, energy transfer to a green fluorescent protein (GFP) and emission of green fluorescence 2. The GFP from jellyfish (*Aequorea victoria*) and its variants have become one of the most popular fluorescent indicators for protein localization, as biosensors for cellular events, and for gene expression studies 3–6. In particular, pH-sensitive GFP variants have enabled the assessment of the intracellular pH change in living cells 7–10. Recent studies have reported GFP-based ratiometric pH-sensitive probes such as pHluorin 9, deGFP 11, E^2^GFP 12, pHred 13, pHusion 14 and others 15–18, which have dual -excitation or -emission maxima and exhibit differential fluorescence excitation or emission in response to changes in pH. The utility of such ratiometric measurement was demonstrated by the fact that it can reduce or eliminate distortions of data caused by nonuniform concentrations and distributions of the probe, nonstability of excitation light, and photobleaching within cells or between groups of cells.

The sea cactus *Cavernularia obesa* is a bioluminescent anthozoan that emits green light (509 nm maximum) *in vivo* but blue light (490 nm maximum) in cell lysate 19. This difference in wavelength is caused by bioluminescence resonance energy transfer owing to the presence of GFP 20. We examined the *C. obesa* bioluminescence system using isolation of the GFP gene, characterization of the native dimeric GFP, and preparation and characterization of monomeric mutants of this GFP. Our results show that the *C. obesa* GFP (CoGFP) has pH-sensitive and ratiometric dual-excitation and -emission properties and also dual-color emission maxima upon single- wavelength excitation. A remarkable feature of CoGFP is its ability to fluoresce in an acidic environment down to pH 4. We then used the monomeric mutant to visualize intracellular pH change during the phagocytosis of living cells using single-wavelength excitation fluorescence microscopy and demonstrated this system's ease of use compared with dual-emission fluorescent proteins that require dual-wavelength excitation.

## Materials and methods

### Collection of specimens

Sea cactus (*C. obesa*) specimens were collected at a depth of 5–10 m at the bottom of Nakaumi, a brackish lake in Shimane prefecture, Japan, during October 2007. All samples were dissected and washed with seawater through a depth filter, frozen on dry ice, and stored at –80°C.

### Purification of CoGFP

Superficial coenenchyme tissue (200 g) from 15 individuals was minced with scissors and homogenized by a rotor-stator homogenizer (Polytron PT 1300D, Kinematic AG, Littau, Switzerland) in 500 mL of 25 mM Tris/glycine (pH 8.3) containing 0.1% sodium dodecyl sulfate (SDS) on an ice-water bath. The homogenate was centrifuged at 8,000 ***g*** for 30 min at 4°C, and the crude GFP solution was fractionated by precipitation with 80% ammonium sulfate. The precipitate was centrifuged at 8,000 ***g*** for 30 min at 4°C and suspended with 50 mL of Tris chromatography buffer (TCB; 20 mM Tris/HCl pH 7.0 with 20 mM NaCl). The suspension was fractionated by precipitation with 67% ethanol, and the precipitate was centrifuged at 8,000 ***g*** for 30 min at 4°C and suspended in 50 mL of TCB. The suspension was dialyzed against 5 L of TCB.

The dialyzed sample was subjected to four steps of column chromatography. First, the sample was loaded onto a DEAE Sepharose CL-6B ion-exchange column (2.6 × 7.0 cm, GE Healthcare Bioscience, Buckinghamshire, UK) equilibrated with 20 mM Tris/HCl (pH 7.0) using the ÄKTA explore 10S chromatography system (GE Healthcare Bioscience), and fractionated with an NaCl gradient of 20 to 400 mM (in TCB) at a flow rate of 0.5 mL/min. Fluorescent fractions (visualized by UV irradiation) were collected and dialyzed against 5 L of TCB and concentrated by ultrafiltration with a 30 kDa cut-off filter (Amicon Ultra-15, Millipore, Billerica, MA, USA). Second, the concentrated sample was loaded onto a Sephacryl S-200 high-resolution gel filtration column (2.6 × 56.0 cm, GE Healthcare Bioscience) equilibrated with TCB and fractionated with TCB at a flow rate of 0.5 mL/min. Third, the fluorescent fractions from S-200 chromatography were loaded onto a Mono Q 5/50 ion-exchange column (0.5 × 5.0 cm, GE Healthcare Bioscience) and fractionated with an NaCl gradient of 20 to 400 mM (in TCB) at a flow rate of 0.5 mL/min. The fluorescent fractions were dialyzed against 5 L of TCB and concentrated by ultrafiltration (30 kDa cut-off). Finally, the concentrated sample was loaded onto a Superdex 75 10/300 GL gel-filtration column (1.0 × 30.0 cm, GE Healthcare Bioscience) equilibrated with TCB and fractionated with TCB at a flow rate of 0.5 mL/min.

The partially purified CoGFP was subjected to 10% pseudo-native SDS/PAGE. The discontinuous buffer system consisted of a 3% acrylamide stacking gel with 0.125 mM Tris/HCl (pH 6.8), a 10% acrylamide separating gel with 0.375 mM Tris/HCl (pH 8.9), and an upper- and lower-electrode buffer with 25 mM Tris/glycine (pH 8.3). All components of the system contained 0.1% SDS. The GFP sample in TCB was mixed with an equal volume of sample buffer (125 mM Tris/HCl, pH 6.8, containing 4% SDS and 50 mM dithiothreitol) and loaded onto the gel without boiling to prevent denaturation. After electrophoresis, the GFP band was visualized by UV illumination and excised for determination of amino acid sequence.

### Amino acid sequence analysis

The CoGFP sample was digested with lysyl endopeptidase at 95°C for 20 h at pH 8.5 and separated by reversed-phase high-performance liquid chromatography (Symmetry C_18_ column, Waters, Milford, MA, USA). The major absorbance fraction was subjected to amino acid sequence analysis by the Edman method with the Procise 494HT Protein Sequencing System (GE Healthcare Science).

### cDNA cloning and protein expression

Coenenchyme (1 g) from one individual was crushed in liquid nitrogen and homogenized with a Polytron homogenizer in 10 mL of Isogen (Nippon Gene, Tokyo, Japan), an RNA extraction reagent containing phenol and guanidine thiocyanate. Total RNA was isolated from the homogenate by chloroform extraction, and mRNA was isolated by oligo(dT)-cellulose chromatography using Oligotex-dT30 <Super> (Takara, Ōtsu, Japan).

To obtain the full-length cDNA of CoGFP, the mRNA was used to create a cDNA library, and RNA ligase–mediated rapid amplification of 5′ and 3′ cDNA ends (5′-RACE and 3′-RACE) was performed with the GeneRacer kit (Invitrogen, Carlsbad, CA, USA). Table 1 shows the primer sets for the first PCR and for the nested second and third PCRs for 5′-RACE and 3′-RACE. The 5′- and 3′-ends of cDNA were cloned into the TA cloning vector, pGEM-T Easy Vector System I (Promega, Madison, WI, USA) and sequenced by the 3130xl Genetic Analyzer (Applied Biosystems, Foster, CA, USA) with the BigDye Terminator v3.1 Cycle Sequencing kit (Applied Biosystems). Based on these sequences, primer sets for the first and nested second PCRs for full-length cDNA of GFP were designed (Table 1), and the nested second PCR product of the full-length cDNA was cloned into the TA cloning vector and sequenced. Based on the nucleotide sequence of the full-length cDNA, codon usage of CoGFP coding region was optimized for expression in mammalian cells, and the synthesized GFP coding sequence (accession number AB743882) was cloned in-frame into *Bam*HI/*Eco*RI multicloning sites of the pRSET-B vector (Invitrogen) for bacterial expression.

**Table 1:** A list of PCR primers used in this study. The symbols of H and N denote mixture of base, A, C and G, and A, C, G and T, respectively

No.	Primer name	Sequence
1	COGFP-TTT	5'-ATHCCNGATTATTTTGT-3'
2	COGFP-TTC	5'-ATHCCNGATTATTTCGT-3'
3	COGFP-TCT	5'-ATHCCNGATTACTTTGT-3'
4	COGFP-TCC	5'-ATHCCNGATTACTTCGT-3'
5	COGFP-CTT	5'-ATHCCNGACTATTTTGT-3'
6	COGFP-CTC	5'-ATHCCNGACTATTTCGT-3'
7	COGFP-CCT	5'-ATHCCNGACTACTTTGT-3'
8	COGFP-CCC	5'-ATHCCNGACTACTTCGT-3'
9	COGFP-ATTAA	5'-GAAGGNTTTACNTTTGAAAG-3'
10	COGFP-ACCAA	5'-GAAGGNTTCACNTTCGAAAG-3'
11	COGFP-ATTGA	5'-GAGGGNTTTACNTTTGAGAG-3'
12	COGFP-ACCGA	5'-GAGGGNTTCACNTTCGAGAG-3'
13	GeneRacer3'Primer	5'-GCTGTCAACGATACGCTACGTAACG-3'
14	GeneRacer3'Nested Primer	5'-CGCTACGTAACGGCATGACAGTG-3'
15	COGFP-A-R1	5'-GCTATAGCCGTCTCATGTTGCTCGT-3'
16	COGFP-A-R2	5'-AGCCGTCTCATGTTGCTCGTAGTAG-3'
17	COGFP-A-R3	5'-ATGTTGCTCGTAGTAGTTGCCTTCCTCGAC-3'
18	GeneRacer5'Primer	5'-CGACTGGAGCACGAGGACACTGA-3’
19	GeneRacer5'Nested Primer	5'-GGACACTGACATGGACTGAAGGAGTA-3’
20	COGFP-A-Full-F3	5'-ATTTAGGTGGCTGCGTACAG-3'
21	COGFP-A-Full-F4	5'-ATTTAGGTGGCTGCGTACAGTTAACAC-3'

The CoGFP containing a poly-histidine tag at the N-terminus was expressed in *Escherichia coli* strain JM109 (DE3) (Promega) with the pRSET-B expression system. Cultured cells in 50 mL of Luria–Bertani medium containing ampicillin (100 µg/mL) were resuspended in 20 mL of 50 mM Tris/HCl (pH 8.0) containing 500 mM NaCl and were ultrasonicated on an ice-water bath for 20 min continuously. After centrifugation, the supernatant was applied to a Ni-NTA agarose resin column (Qiagen, Hilden, Germany). The GFP was eluted with 50 mM Tris/HCl (pH 8.0) containing 500 mM NaCl and 200 mM imidazole, and the buffer of the fractionated sample was exchanged with a PD-10 column (GE Healthcare Bioscience) into 50 mM Tris/HCl (pH 8.0) containing 200 mM NaCl.

### Spectroscopy and pH titration

Absorption and fluorescence spectra of CoGFP with a histidine tag at the N-terminus were determined with a U-3010 spectrophotometer (Hitachi, Tokyo, Japan) and a F-2500 fluorescence spectrometer (Hitachi). pH titrations were performed in a series of buffers ranging from pH 4 to pH 8 (citrate-phosphate buffer created with 0.1 M citric acid and 0.2 M dibasic sodium phosphate) and from pH 9 to pH 11 (0.1 mM glycine–NaOH buffer). The quantum yield of fluorescence (Φ) of the GFP samples and that in different pH conditions was calculated relative to samples of enhanced GFP (EGFP) and enhanced blue fluorescent protein (EBFP) of equal optical density (OD = 0.01) and the known values for these proteins (EGFP: Φ  = 0.60 at 488 nm, pH 7; EBFP: Φ = 0.17 at 380 nm, pH 7) 21.

### Mutagenesis

Site-directed and semi-random mutations were introduced as described by Sawano and Miyawaki 22. Random mutagenesis targeted to the protein sub-domain was performed with the GeneMorph II EZClone Domain Mutagenesis kit (Stratagene, La Jolla, CA, USA).

### Monomerization assay

Size-exclusion chromatography was used to assess monomerization of mutagenized CoGFPs. GFP (50 μL, 1 mg/mL) was loaded onto a Superdex 200 prep-grade column (7 × 50 cm; GE Healthcare Science) with the elution buffer (10 mM Tris/HCl pH 8.0 containing 150 mM NaCl) at a flow rate of 0.83 mL/min. A gel-filtration standard (Bio-Rad, Hercules, CA, USA) was used as a molecular size marker. The mutagenized CoGFP samples were also assessed by pseudo-native SDS/PAGE (14% acrylamide) without boiling the samples to prevent denaturation.

### Fluorescence microscopy

HeLa (ECACC, Salisbury, UK) or RAW264.7 (ATCC, Manassass, VA, USA) cells were cultured on a 35-mm glass-bottom dish in Dulbecco's modified Eagle's medium (Invitrogen) containing 10% fetal bovine serum. The monomeric CoGFP (variant-0) and EGFP genes were inserted into a mammalian expression vector pcDNA 3.1 (Invitrogen). After transfection using Lipofectamine 2000 (Invitrogen), HeLa cells were washed with Dulbecco's phosphate-buffered saline [pH 7.2; PBS(–)] and fixed in 4% paraformaldehyde in 100 mM PBS (pH 7.4). Fluorescence images were captured with an IX-70 inverted microscope (Olympus, Tokyo, Japan) equipped with a UPLNAPO 40X objective lens, a WU mirror unit (330–385 nm band-pass excitation filter, 400 nm dichroic mirror, 420 nm long-pass emission filter), a NIBA mirror unit (470–490 nm band-pass excitation filter, 505 nm dichroic mirror, 510–550 nm band-pass emission filter), and a DP70 color CCD camera (Olympus). Then, HeLa cells were washed with a citrate-phosphate buffer (pH 4.0), and fluorescence images were captured with the WU and NIBA mirror units.

*Escherichia coli* strain JM109(DE3) expressing mutagenized CoGFP (variant-0) and EGFP was subjected to phagocytosis by mouse macrophage (RAW264.7) cells, and fluorescence images were captured with the WU and NIBA mirror units after change of the medium to Dulbecco's PBS(–).

## Results

### Amino acid sequence and primer design

We initially tried to clone CoGFP gene using PCR with primer sets designed based on *Renilla* and *Ptilosarcus* GFP genes (accession numbers AY015996, AF372525 and AY015995), but we were not successful. Thus, we designed suitable PCR primers based on the amino acid sequence of the purified CoGFP. This led to identification of a 22-residue peptide: I-P-D-Y-F-V-Q-S-F-P-E-G-F-T-F-E-R-T-L-S-F-E. We then designed twelve 3′-RACE primers based on this amino acid sequence (Table 1). Primer sequences for the 3′-RACE, Nos 1–8 and No. 9–12, were deduced from the amino acid sequences IPDYFV and EGFTFER, respectively, of the GFP. 5′-RACE primers Nos 15–17 and full-length primers Nos 20 and 21 were designed after sequencing of products from 3′-RACE and 5′-RACE, respectively. Other primers (Nos 13, 14, 18, 19) were from the GeneRacer kit.

### cDNA cloning and sequencing

The first and nested second PCRs for 3′-RACE were successful using primer sets Nos 2 and 13 and Nos 10 and 14, respectively, against the cDNA library. The nested second PCR product yielded a single band upon agarose gel electrophoresis (data not shown). For 5′-RACE, the first PCR and the nested second and third PCRs were successful using primer sets Nos 15 and 18, Nos 16 and 19, and Nos 17 and 19, respectively. The nested third PCR product yielded a single band upon agarose gel electrophoresis. Based on the sequences obtained from 3′- and 5′-RACE, the full-length cDNA was obtained by the first and nested second PCRs using primer sets Nos 20 and 13 and Nos 21 and 14, respectively. The nested second PCR product yielded a single band upon agarose gel electrophoresis.

**Figure 1:**
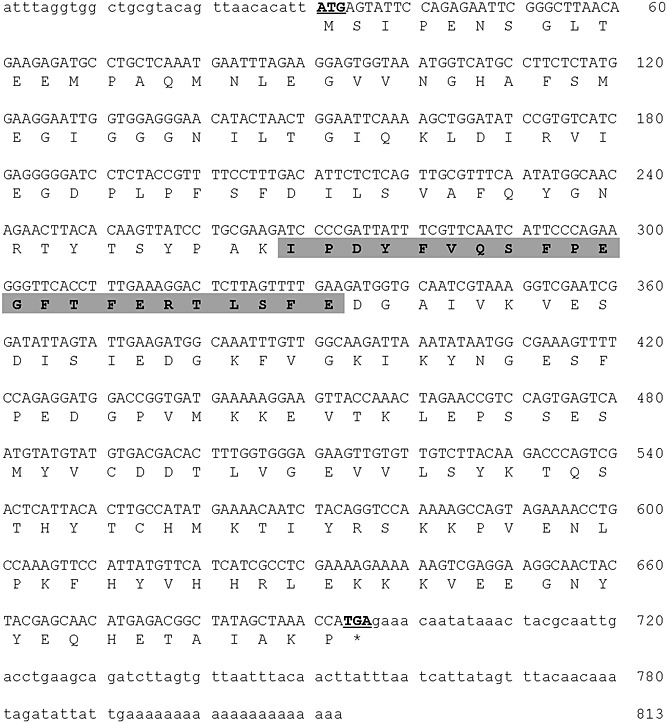
cDNA nucleotide sequence and deduced amino acid sequence of *Cavernularia obesa* GFP (CoGFP). In the nucleotide sequence, upper case letters indicate the ORF, lower case letters indicate untranslated regions, and underlined upper case letters indicate the start and stop codons. The light-gray shading indicates a sequence identical to that determined by Edman degradation of the purified protein.

Figure 1 shows that the sequence of the isolated cDNA was 813 bp with a 30-bp 5′-untranslated region (UTR) upstream from the first start codon (ATG) and a 117-bp 3′-UTR downstream that was between the stop codon (TGA) and the terminal poly(A) (AB743881). The open reading frame (ORF) contained 663 bp, which encoded 221 amino acid residues. The amino acid sequence of the 22 residues used for primer design corresponded to residues 80–101, highlighted in the gray box (Fig. 1).

**Figure 2:**
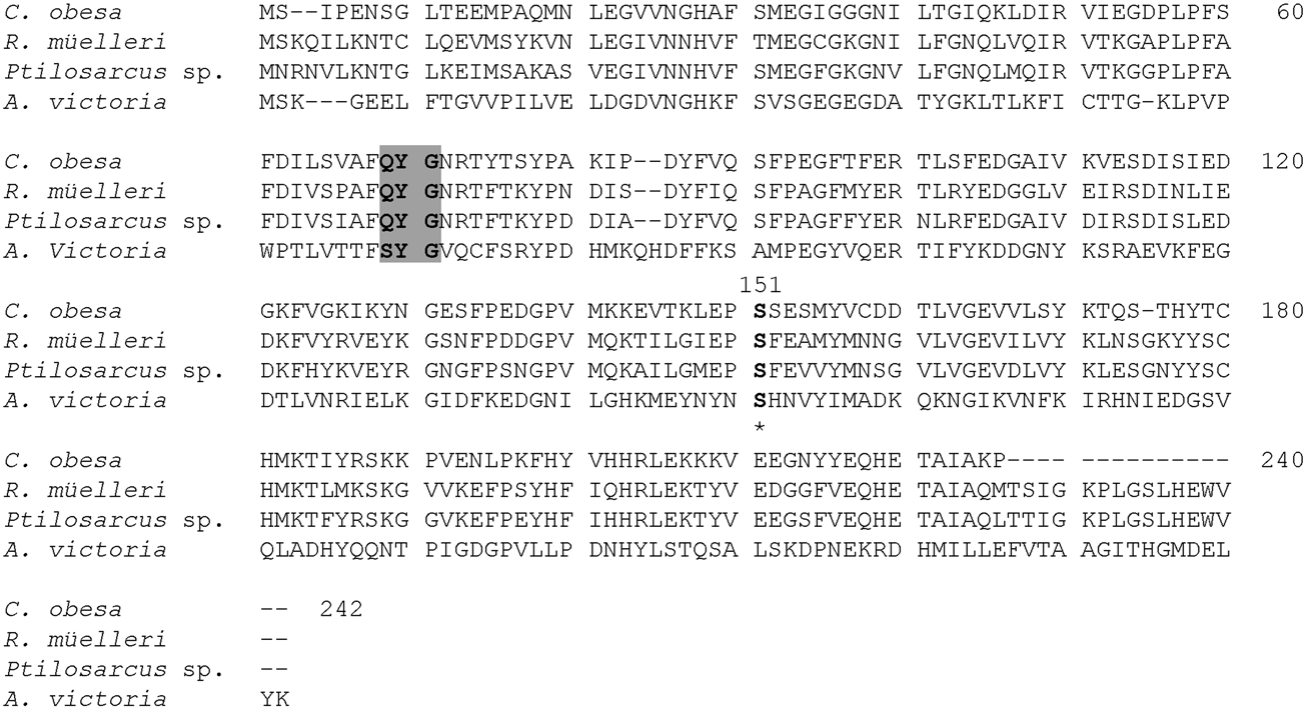
Comparison of the amino acid sequences of GFPs from *Cavernularia obesa*, *Renilla mülleri*, *Ptilosarcus* sp., and *Aequorea victoria*. Hyphens indicate gaps inserted to optimize the overall sequence alignment, the light-gray shading indicates the chromophore-forming tripeptide, and the asterisk indicates the Ser at position 147 of *A. victoria* GFP.

Figure 2 shows a comparison of this amino acid sequence with GFPs of three related species, *Renilla mülleri* (AY015996), *Ptilosarcus* sp. ( AY015995), and *A. victoria* (M62654). The chromophore-forming tripeptide of the three GFPs (*Cavernularia*, *Renilla* and *Ptilosarcus* belonging to the order Pennatulacea) is Gln–Tyr–Gly located at aligned residues 69–71, but that is Ser–Tyr–Gly in *Aequorea* GFP. The amino acid sequence identity among Pennatulacea GFPs and that between CoGFP and *Aequorea* GFP was 58.8% and 25.1%, respectively.

### Subunit and spectral properties

**Figure 3:**
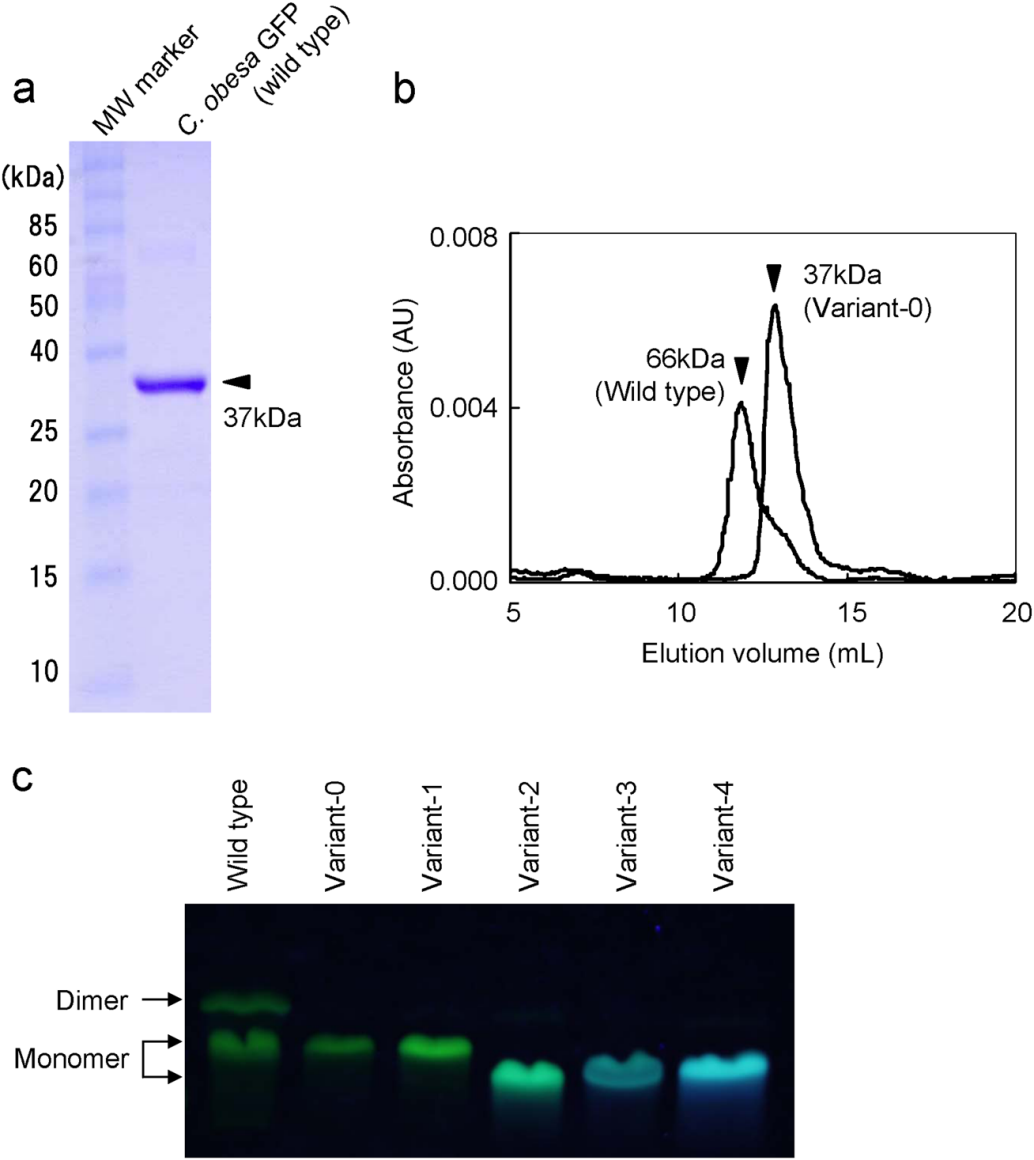
Apparent molecular mass of wild-type CoGFP and of five CoGFP variants. Molecular mass determination of wild-type CoGFP by 10% SDS/PAGE (a), size-exclusion chromatography of the wild-type and variant-0 CoGFPs (b) and identification of monomeric and dimeric forms in wild-type and variants by 14% pseudo-native SDS/PAGE under 362 nm excitation (c).

We determined that the molecular mass of CoGFP with a histidine tag at the N-terminus was 37 kDa based on 10% SDS/PAGE but was 66 kDa based on size-exclusion chromatography (Fig. 3a,b). Furthermore, use of 14% pseudo-native SDS/PAGE led to two bands (Fig. 3c). Taken together, these results indicated that the native CoGFP is a dimer.

**Figure 4:**
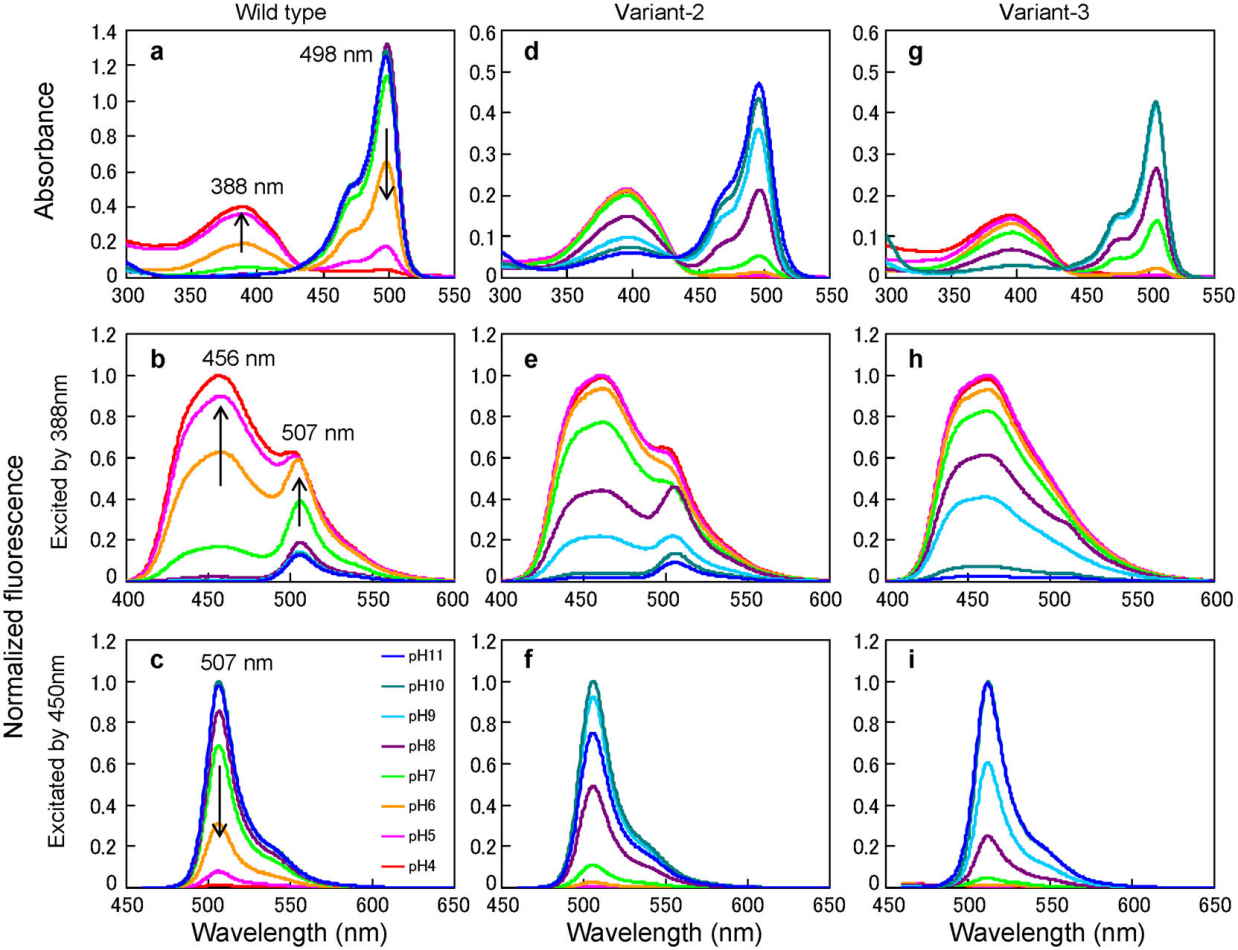
Absorption spectra (a,d,g) and normalized fluorescence spectra during 388 nm excitation (b,e,h) and 450 nm excitation (c,f,i) at pH 4 to 11 (mean of three replicates) in wild-type CoGFP (a,b,c), variant-2 CoGFP (d,e,f), and variant-3 CoGFP (g,h,i). Arrows show the effect of decreasing pH.

**Figure 5:**
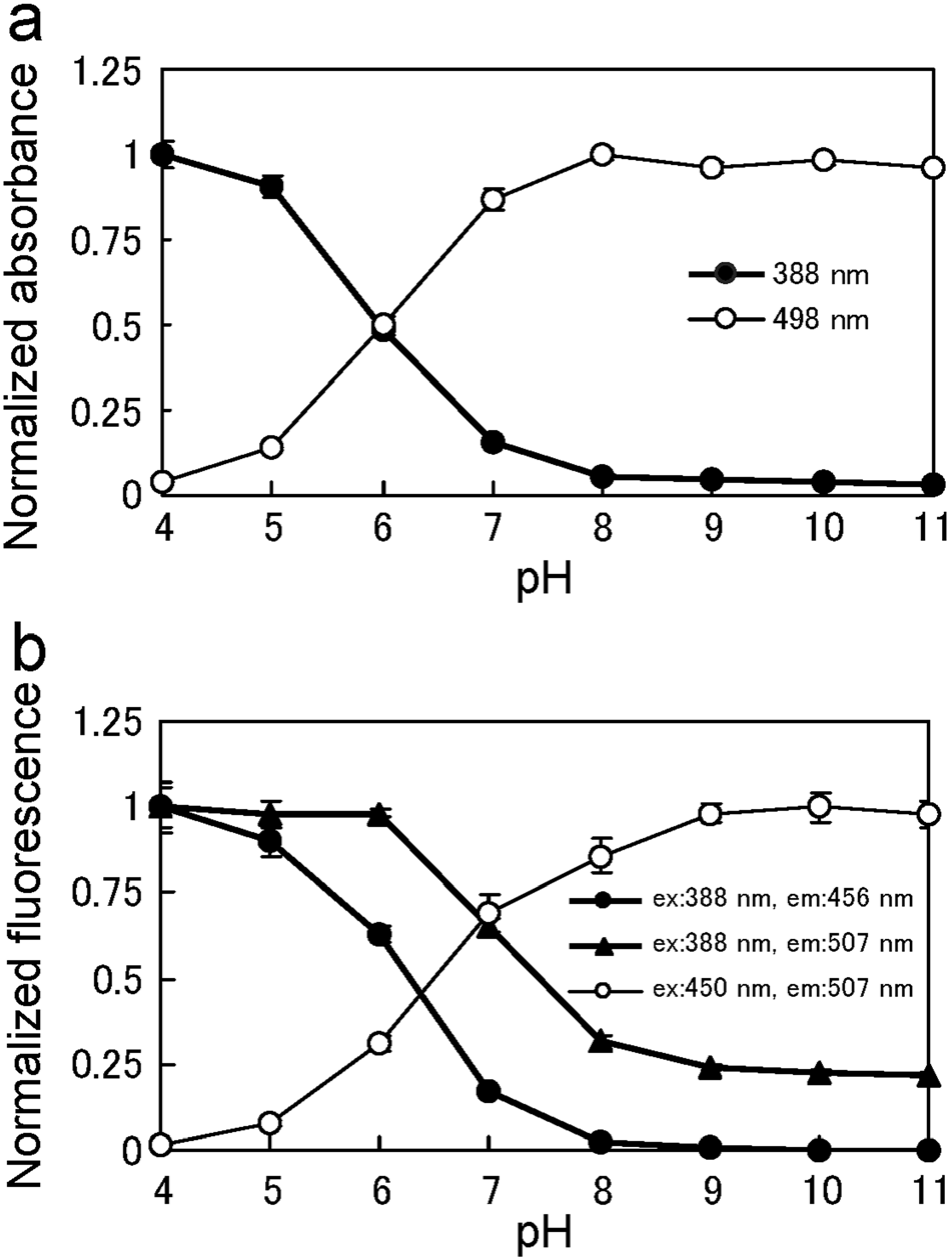
Effect of pH on the absorption maxima (388, 498 nm) (a) and fluorescence maxima (456, 507, 388 nm excitation; 507, 450 nm excitation) of wild-type CoGFP (b) (mean ± SD, *N* = 3).

The absorption spectrum of CoGFP indicated maxima at 388 and 498 nm, and a slight shoulder at 471 nm (Fig. 4a). As pH decreased, the intensity of the 388 nm peak increased and that of the 498 nm peak decreased. The isosbestic point was 435 nm at all tested pH values. Table 2 shows the molar absorption coefficients (ε) of the 388 and 498 nm peaks at different pH values. Figure 5(a) shows the effect of pH on absorption at 388 and 498 nm, and indicates that absorption was ratiometric and their p*K*_a_ values were the same, i.e. 6.0.

**Table 2:** Spectral properties of the *Cavernularia obesa* GFP and its variants

	pH	λ_abs_/λ_em_ (nm)	ε (M^-1^ cm^-1^)	Φ
Wild-type	5	388/456, 502	47,200 (388 nm)	0.28 (380 nm)
		498/506	23,500 (498 nm)	ND
	7	392/457, 506	8,000 (388 nm)	ND
		499/507	147,300 (498 nm)	0.87 (488 nm)
	9	389/506	2,300 (388 nm)	0.30 (380 nm)
		498/507	163,600 (498 nm)	1.00 (488 nm)
Variant-0	5	390/458, 502	56,500 (388 nm)	0.32 (380 nm)
		498/506	30,800 (498 nm)	ND
	7	392/458, 506	11,000 (388 nm)	ND
		498/507	218,900 (498 nm)	0.73 (488 nm)
	9	390/506	3,300 (388 nm)	0.28 (380 nm)
		498/507	232,000 (498 nm)	1.00 (488 nm)
Variant-1	5	393/458	26,600 (388 nm)	0.44 (380 nm)
		499/507	4,800 (498 nm)	ND
	7	395/460, 504	11,900 (388 nm)	ND
		500/507	68,400 (498 nm)	0.88 (488 nm)
	9	398/504	3,400 (388 nm)	0.29 (380 nm)
		500/507	96,500 (498 nm)	1.00 (488 nm)
Variant-2	5	396/462	20,000 (388 nm)	0.28 (380 nm)
		ND/ND	ND	ND
	7	395/462	17,000 (388 nm)	ND
		497/506	2,300 (498 nm)	ND
	9	398/460, 504	8,900 (388 nm)	0.15 (380 nm)
		496/506	24,700 (498 nm)	0.98 (488 nm)
Variant-3	5	396/462	25,800 (388 nm)	0.33 (380 nm)
		ND/ND	ND	ND
	7	396/463	24,000 (388 nm)	ND
		ND / ND	ND	ND
	9	396/461	11,600 (388 nm)	0.39 (380 nm)
		506/512	43,900 (498 nm)	0.27 (488 nm)
Variant-4	5	397/461	35,200 (388 nm)	0.50 (380 nm)
		ND/ND	ND	ND
	7	397/461	36,200 (388 nm)	ND
		ND/ND	ND	ND
	9	397/462	29,200 (388 nm)	0.44 (380 nm)
		503/509	21,700 (498 nm)	1.00 (488 nm)

λ_abs_, absorption maxima; λ_em_, fluorescence maxima excited by the λ_abs_ light; ε, molar absorption coefficient at 388 and 498 nm; Φ, fluorescence quantum yield at 388 and 468 nm; ND, not determined (*N* = 3).

Figure 4(b) and (c) show the fluorescence spectra of CoGFP. Excitation at 388 nm led to fluorescence with maxima at 456 and 507 nm, and the intensity of these peaks increased as pH decreased (Fig. 4b). By contrast, under excitation at 450 nm, fluorescence at 507 nm decreased as pH decreased (Fig. 4c). Figure 5(b) shows the effect of pH on fluorescence. Under excitation at 388 nm, fluorescence emission at 456 nm (p*K*_a_ = 6.5) was more sensitive to acidity than fluorescence at 507 nm (p*K*_a_ = 7.5). Under 450 nm excitation, fluorescence at 507 nm was ratiometric to that at 456 and 507 nm under 388 nm excitation, and the pKa value of fluorescence at 507 nm was p*K*_a_ = 6.5.

### Monomeric and pH-sensitive variants of CoGFP

Introduction of any of five mutations (S129G, C154S, D156G, K204I and N209Y; variant-0: AB743883) resulted in monomerization of CoGFP (Fig. 3b,c). The fluorescence of variant-0 was significantly greater than that of wild-type (Table 2). In particular, the ε at 388 nm increased from 47,200 (wild-type) to 56,500 at pH 5, and the ε at 498 nm increased from 163,600 to 232,000 at pH 9. The fluorescence Φ under 380 nm excitation increased from 0.28 (wild-type) to 0.32 at pH 5, but the fluorescence Φ under 488 nm excitation was the same (1.00) at pH 9 (Table 2). Furthermore, variant-0 had a faster chromophore maturation time at 37°C than wild-type (data not shown). The wavelength maxima of absorption and fluorescence of variant-0 were nearly identical with those of wild-type (data not shown).

Substitution at residue 147 (corresponding to position 151 of the aligned sequences in Fig. 2) in variant-0 modified the pH sensitivity of fluorescence. In particular, substitution of this Ser with Thr (S147T, variant-1: AB743884), Gly (S147G, variant-2: AB743885), Cys (S147C, variant-3: AB743886) or Ala (S147A, variant-4: AB743887) led to increased blue fluorescence (456 nm maximum) and decreased green fluorescence (507 nm maximum) following 388 nm excitation in turn relatively (Fig. 4b,e,h). Therefore, the spectral data of variants 2 and 3 were shown as representatives of the variants. Furthermore, we confirmed the monomeric nature of these variants by pseudo-native SDS/PAGE (Fig. 3c). Table 2 shows additional spectral properties of these variants.

**Figure 6:**
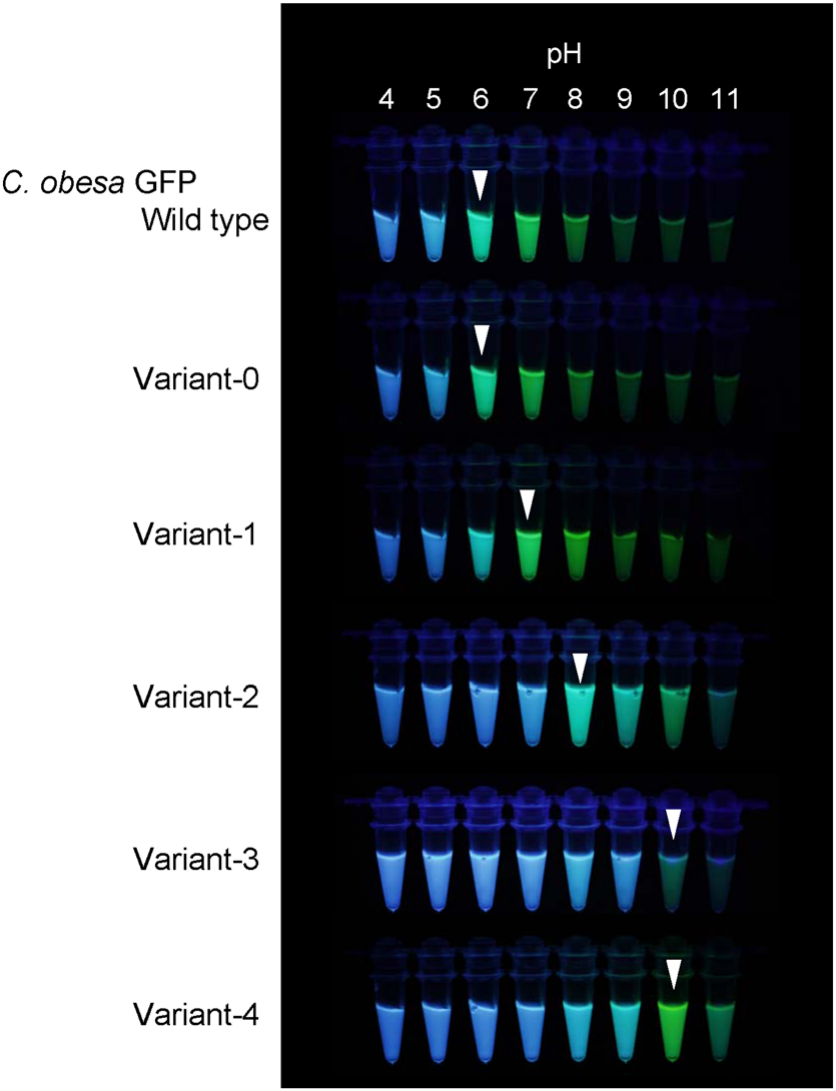
Fluorescence of wild-type CoGFP and of five CoGFP variants excited by 362 nm using a handy UV-lamp in Eppendorf tubes at pH 4 to 11. Each arrowhead indicates the transition from blue to green fluorescence.

**Figure 7:**
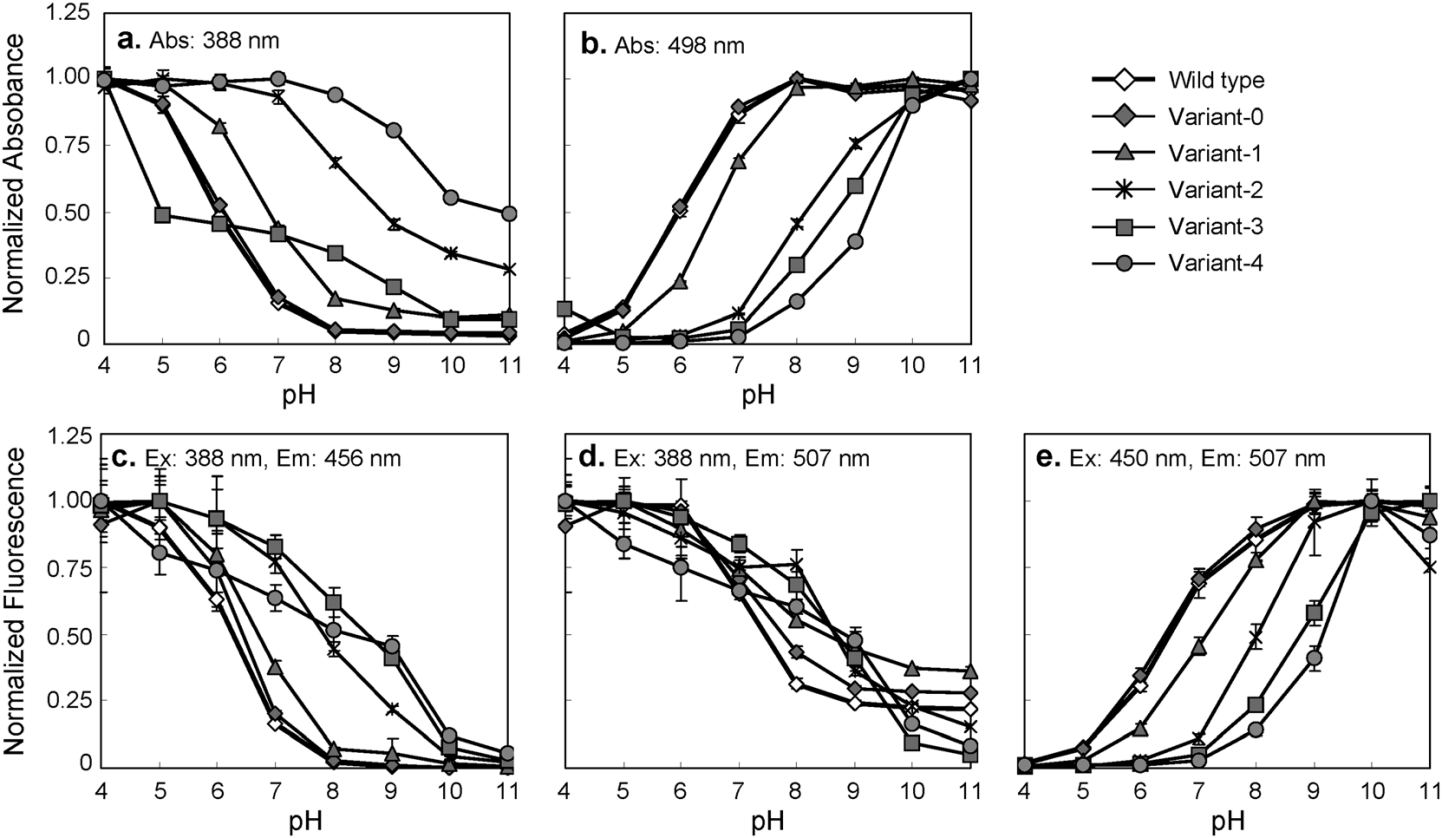
Effect of pH on absorption (Abs.) at 388 nm (a) or 498 nm (b) and fluorescence emission (Em.) at 456 nm (388 nm excitation, Ex.) (c), 507 nm (388 nm excitation) (d), and 507 nm (450 nm excitation) (e) of wild-type CoGFP and of five variant CoGFPs (mean ± SD, *N* = 3).

Figure 6 shows a photograph of fluorescence of the wild-type and variant CoGFPs under 362 ± 4 nm excitation using a handy UV-lamp (SLUV-8, As One, Tokyo, Japan) at pH 4–11. The transition point from blue to green fluorescence was moved from pH 6 (wild-type) to pH 10 (variants 3 and 4) corresponding to an increase in the p*K*_a_ of the blue fluorescence (456 nm, 388 nm excitation) and green fluorescence (507 nm, 388 nm excitation) from 6.5 to 8.5 and from 7.5 to 9.0, respectively (Fig. 7c,d).

### Fluorescence microscopy

**Figure 8:**
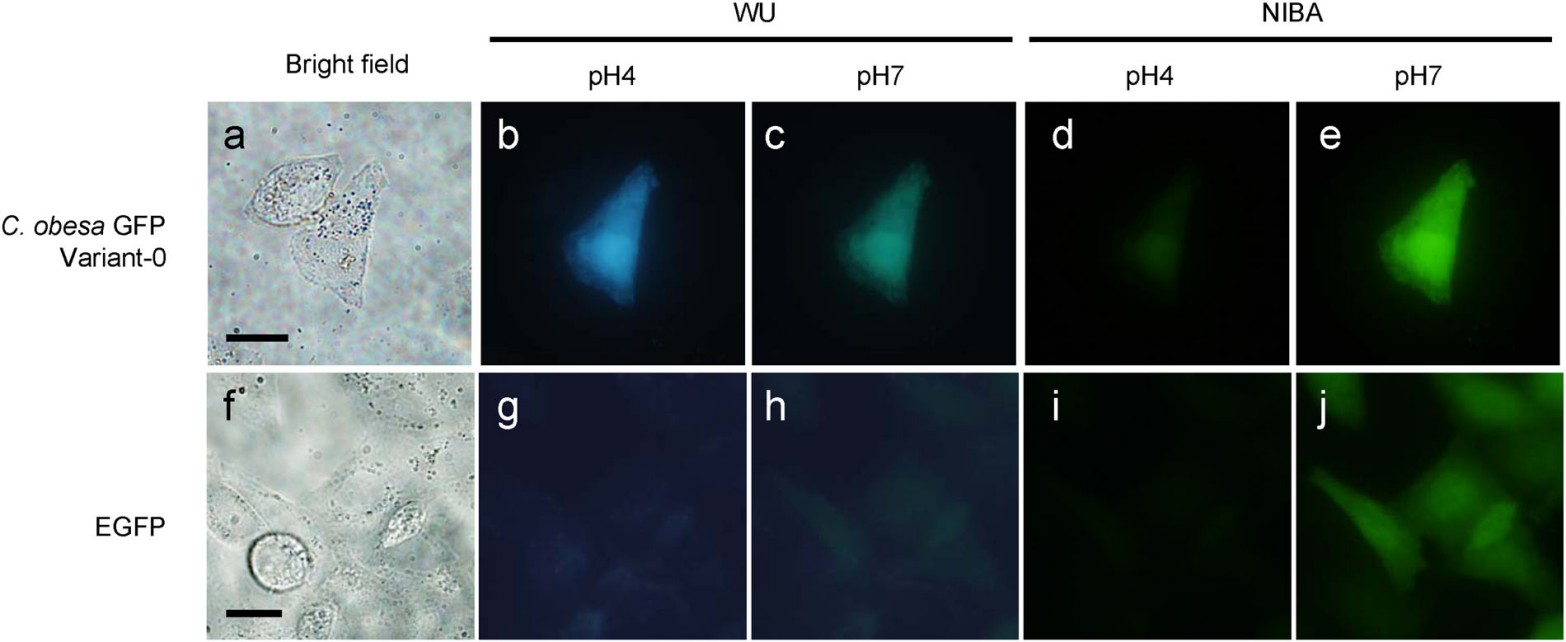
Bright-field (a,f) and fluorescence (b–e,g–j) images of HeLa cells transiently expressing variant-0 CoGFP and EGFP. Fluorescence images were captured by WU and NIBA mirror units at pH 4 and pH 7. Scale bars, 20 µm.

We used fluorescence microscopy to observe HeLa cells fixed by paraformaldehyde that expressed variant-0 CoGFP and EGFP (Fig. 8). A direct comparison of CoGFP and EGFP in the fluorescence microscope is not possible because the excitation and emission spectra of these GFPs are different. Therefore, we use two different filter sets, optimized for either of them; WU for CoGFP and NIBA for EGFP. Under UV light excitation (330–385 nm) using the WU mirror unit, variant-0 emitted blue and cyan fluorescence at pH 4 and pH 7, respectively (Fig. 8b,c). The image at pH 7 appeared to provide only a slight decrease in fluorescence intensity with cyanic as unexpected from *in vitro* experiments (Fig. 6). This could be explained as an effect of incomplete intracellular pH change. As expected, no EGFP fluorescence was visible owing to a mismatch of the excitation and emission wavelengths in this system (Fig. 8g,h). Under blue-light excitation (470–490 nm) using the NIBA mirror unit, variant-0 emitted faint green fluorescence at pH 4 and strong green fluorescence at pH 7 (Fig. 8d,e). Again, as expected, EGFP emitted no fluorescence at pH 4 owing to the lack of fluorescence activity in an acidic environment (Fig. 8i), but emitted substantial fluorescence at pH 7 (Fig. 8j).

**Figure 9:**
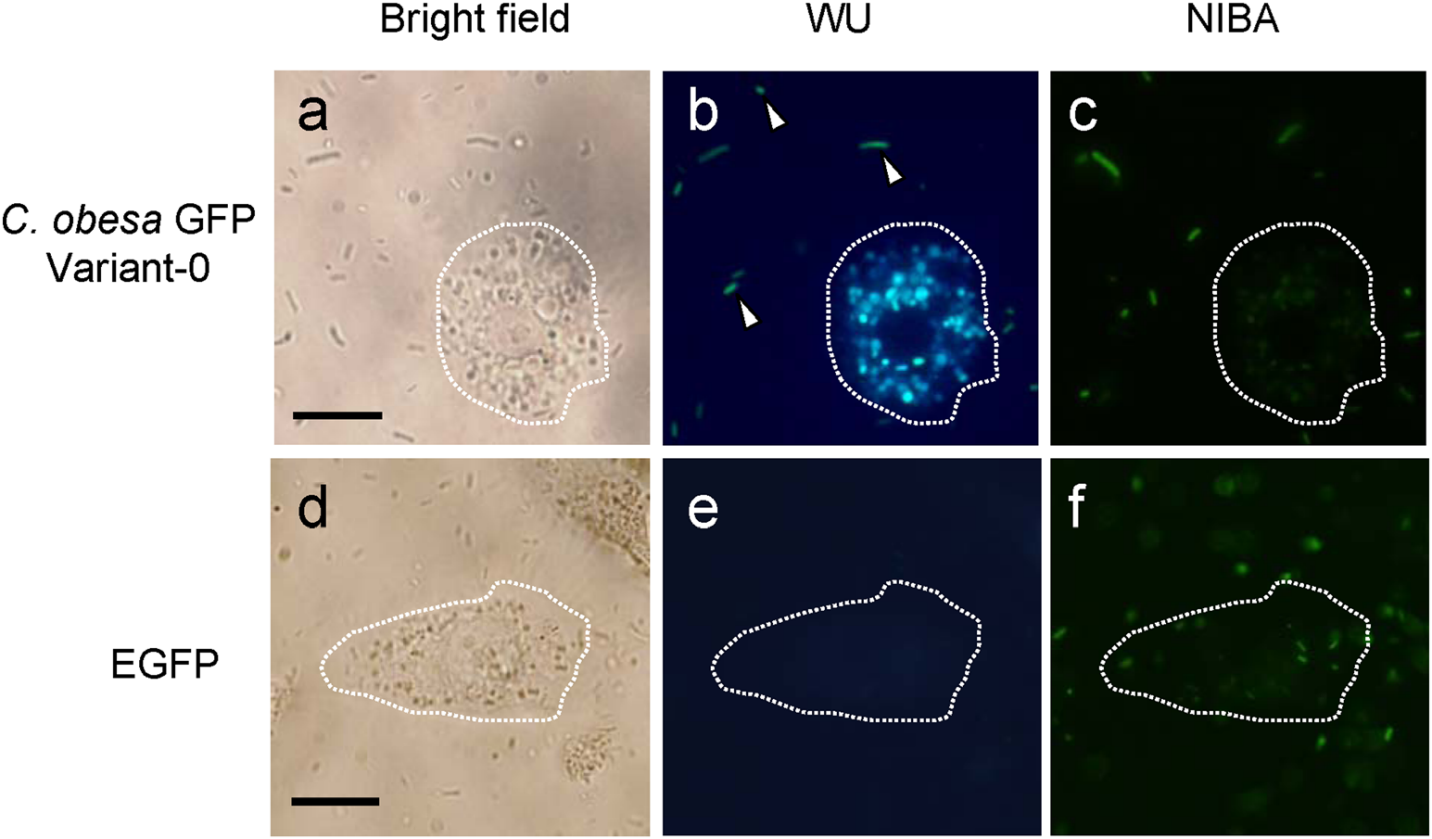
Bright-field (a,d) and fluorescence (b,c,e,f) microscopy of macrophages (RAW264.7) feeding on *E. coli* that expressed variant-0 CoGFP and EGFP. Fluorescence images were captured by the WU and NIBA mirror units. The dotted line in each image outlines a RAW264.7 cell, and arrowheads indicate *E. coli* cells. Scale bars, 20 µm.

Finally, we tested the applicability of variant-0 for living cells studies by observing cells of a mouse macrophage line (RAW264.7) that were feeding on *E. coli* expressing variant-0 and EGFP (Fig. 9). Under UV light excitation with the WU mirror unit, *E. coli* cells with variant-0 within the macrophage phagosomes emitted blue fluorescence, whereas cells outside the macrophage emitted green fluorescence (Fig. 9b) owing to the pH difference of these two regions. In addition, the blue cells that had been incorporated into phagosomes had rod-to-round shapes, whereas the green cells outside the macrophage were rod-shaped bacilli. As expected, *E. coli* expressing EGFP emitted no observable fluorescence within or around RAW264.7 cells owing to a mismatch of the excitation and emission wavelengths in this system (Fig. 9e). Under blue-light excitation with the NIBA mirror unit, we observed green *E. coli* expressing variant-0 within and around RAW264.7 cells (Fig. 9c), but images of the *E. coli* incorporated into RAW264.7 cells were faint as was observed in the acidic environment. For EGFP, green *E. coli* were observed around RAW264.7 cells as expected (Fig. 9f); note that green cells that appear to be inside the RAW264.7 cells were actually on the RAW264.7 surface.

## Discussion

A remarkable feature of CoGFP is its ability to fluoresce in an acidic environment down to pH 4, and we created several variants of CoGFP that, upon excitation at 388 nm, transition from blue to green fluorescence at different pH values owing to alterations in the p*K*_a_ (Figs 6 and 7c,d). GFP-based pH indicators which work at the most acidic environment are E^2^GFP 12 and pHusion 14 at pH 4.5. However, CoGFP works even at pH 4. Furthermore, it shows unique spectral property that two fluorescence peaks (456 and 507 nm) are observed by single-wavelength excitation (388 nm), and ratio of the two peaks varies in pH condition (Fig. 4b,e,h). Therefore, CoGFP indicates pH conditions as coloration simply using a color CCD camera without ratiometric calculation. This is very suitable for conventional wide-field fluorescence microscopy with UV excitation and long-pass emission filters. pHusion also emits two fluorescent peaks, but it requires a dual-excitation and emission system, because the pH sensor is constructed by two different florescent proteins, EGFP and monomeric red fluorescent protein (mRFP1) 14. The spectral property of E^2^GFP is also unique in that one fluorescent peak is observed and its peak shifts depending on the pH. However, the amount of peak sift is narrow (10 – 16 nm) 12. Our experiments indicate that CoGFP variants are suitable as pH indicators in cell biology studies. In particular, variant-0 remained fluorescent even when exposed to the acidic (pH ∼ 5) 23 environment inside phagosomes of RAW264.7 macrophages (Fig. 9). This CoGFP emitted blue fluorescence at low pH (in phagosomes) and green fluorescence when it was outside of macrophages. Thus, the variants of CoGFP have potential for use as *in vivo* pH indicators in that they require only a single long-pass emission filter with a single-light excitation system, which is simpler than the system required for dual-emission fluorescent proteins that require dual-excitation.

The absorption spectrum of CoGFP is sensitive to pH, in that the 388 nm absorption peak increases and the 498 nm absorption peak decreases as pH declines. This pH dependency is presumably due to changes in the ionization state of a phenolic hydroxyl group in the chromophore, as in *Aequorea* GFP 3. In contrast to CoGFP, *Aequorea* GFP does not fluoresce when the chromophore is in a neutral state (395 nm absorption peak) owing to rapid and complete transition from the neutral to the ionized state by 395 nm light excitation; green fluorescence only occurs from the ionized state following excitation at 480 nm 3–24. For CoGFP, blue (456 nm) and green (507 nm) fluorescence both occur upon excitation at 388 nm, and green fluorescence also occurs upon excitation at 498 nm. Therefore, the blue fluorescence is from the neutral state and the green fluorescence is from the ionized state of the chromophore owing to the incomplete transition from the neutral to the ionized state, and the transition equilibrium depends on the pH.

Hanson *et al*. 11 constructed dual-emission GFP variants (S65T and H148C and/or T203C) from *Aequorea* GFP that were sensitive to pH. Analysis of the structures of these mutants indicated that residues 143–150 undergo remarkable rearrangement upon a change in pH; the authors proposed a novel proton relay model for excited-state proton transfer that explains the dual-wavelength emission of this protein 11–25. According to this model, at low pH, the structure does not contain a hydrogen bond network to support the rapid transfer of a proton from the excited state of the neural chromophore to a suitable acceptor, and this leads to blue fluorescence. At high pH, backbone rearrangements induced by changes in the hydrogen bond network permit proton transfer from the excited state of the neutral chromophore to bound water molecules and bulk solvent via Ser147 (151 in Fig. 2), and this leads to green fluorescence from the ionized chromophore. For variant-1 to variant-4 of CoGFP (S147T/C/A/G together with monomerized substitutions of variant-0, S129G, C154S, D156G, K204I, and N209Y), there is increased blue fluorescence upon excitation at 388 nm and a corresponding decrease in green fluorescence (Fig. 4e,h). However, the substitution of Ser147 with other amino acids (except Thr) in the wild-type CoGFP yielded proteins that were not fluorescent (data not shown; note that the fluorescence spectral properties of the Thr variant were the same as those of variant-1). Although position 147 (151 in Fig. 2) in CoGFP appears to be crucial in the transition between the neutral and ionized states of the chromophore, structural analysis is needed to confirm the data and to provide a more complete understanding of the mechanism of dual-color emission in CoGFP.
